# Nanoencapsulation of ultra-small superparamagnetic particles of iron oxide into human serum albumin nanoparticles

**DOI:** 10.3762/bjnano.5.235

**Published:** 2014-11-27

**Authors:** Matthias G Wacker, Mahmut Altinok, Stephan Urfels, Johann Bauer

**Affiliations:** 1 Fraunhofer-Institute for Molecular Biology and Applied Ecology, Project group for Translational Research and Pharmacology, D-60438 Frankfurt, Germany; 2 Goethe-University, Institute of Pharmaceutical Technology, D-60438 Frankfurt, Germany; 3 Technische Universität Darmstadt, Technical Chemistry, D-64287 Darmstadt, Germany; 4 Merck KGaA, D-64287 Darmstadt, Germany

**Keywords:** diagnostics, HSA, nanoencapsulation, nanoparticles, USPIO

## Abstract

Human serum albumin nanoparticles have been utilized as drug delivery systems for a variety of medical applications. Since ultra-small superparamagnetic particles of iron oxide (USPIO) are used as contrast agents in magnetic resonance imaging, their encapsulation into the protein matrix enables the synthesis of diagnostic and theranostic agents by surface modification and co-encapsulation of active pharmaceutical ingredients. The present investigation deals with the surface modification and nanoencapsulation of USPIO into an albumin matrix by using ethanolic desolvation. Particles of narrow size distribution and with a defined particle structure have been achieved.

## Introduction

Over the past decades, nanocarriers have been utilized for a variety of different applications. In the area of pharmaceuticals these versatile drug delivery devices enabled the directed transport of drug substances to specific tissues after modification of the particle surface with drug targeting ligands such as antibodies [1–2] and other proteins [3–4].

Aside the specific binding affinity, drug targeting is based on a passive accumulation mechanism that is controlled by particle size and surface characteristics of the colloids. Particles ranging in size between 50 and 300 nm accumulate in solid tumors due to the enhanced permeability and retention effect [5]. While circulating through the blood stream, these colloids undergo an opsonization by the immune system followed by endocytosis into macrophages. Particles of greater diameters are rapidly cleared from the plasma and smaller colloidal carriers are eliminated through the kidney [6]. With increasing circulation time, the extent of passive accumulation into the target tissue increases significantly [7].

The polymeric matrices used in drug formulations for intravenous injection have to comply with highest safety standards due to the systemic exposition of patients with the carrier [6]. Human serum albumin (HSA) represents a promising candidate that binds a wide range of compounds with different physicochemical characteristics. In 2007, with Abraxane^®^, a first polymeric nanoparticle formulation consisting of this material has been approved by the Food and Drug Administration of the United States of America and the European Medicines Agency [6]. These nanoparticles demonstrated an outstanding potential for drug delivery applications due to a long circulation time and enhanced uptake into tumor tissues by specific transporters [8].

In the present study, nanoparticles consisting of HSA were formed by ethanolic desolvation [9]. These nanocarriers were used matrix system for the encapsulation of USPIO. USPIO have been efficiently applied as contrast agents for magnetic resonance imaging and allow the tracking of nanoparticles in vivo [10–11]. Nanocarriers of this size range have been modified by adsorptive binding or incorporation of drug substances earlier [12–14]. Unspecific interactions with the matrix material enabled the binding of drugs such as obidoxime [13], or the binding of hydrophilic complexes of poorly soluble molecules [14].

Since the HSA molecule is negatively charged during desolvation process, positively charged compounds demonstrate a high affinity to the matrix material [13]. Therefore, magnetite nanoparticles have been modified in order to increase charge–charge interactions between USPIO and the matrix material. USPIO HSA hybrid particles of high iron load and narrow size distribution have been achieved.

## Results and Discussion

Prior to the modification of the particle surface by ion layer technique, the structure and magnetization of USPIO were investigated. Afterwards, the surface-modified positively charged core particles were embedded into the HSA matrix by ethanolic desolvation of the protein [9].

By adjusting the reaction conditions of the desolvation procedure, particles of optimal size distribution and surface properties for drug targeting applications have been achieved. The USPIO load, particle diameter, size distribution, particle shape, and surface charge have been investigated.

### Characterization of magnetite core particles

**Figure 1:**
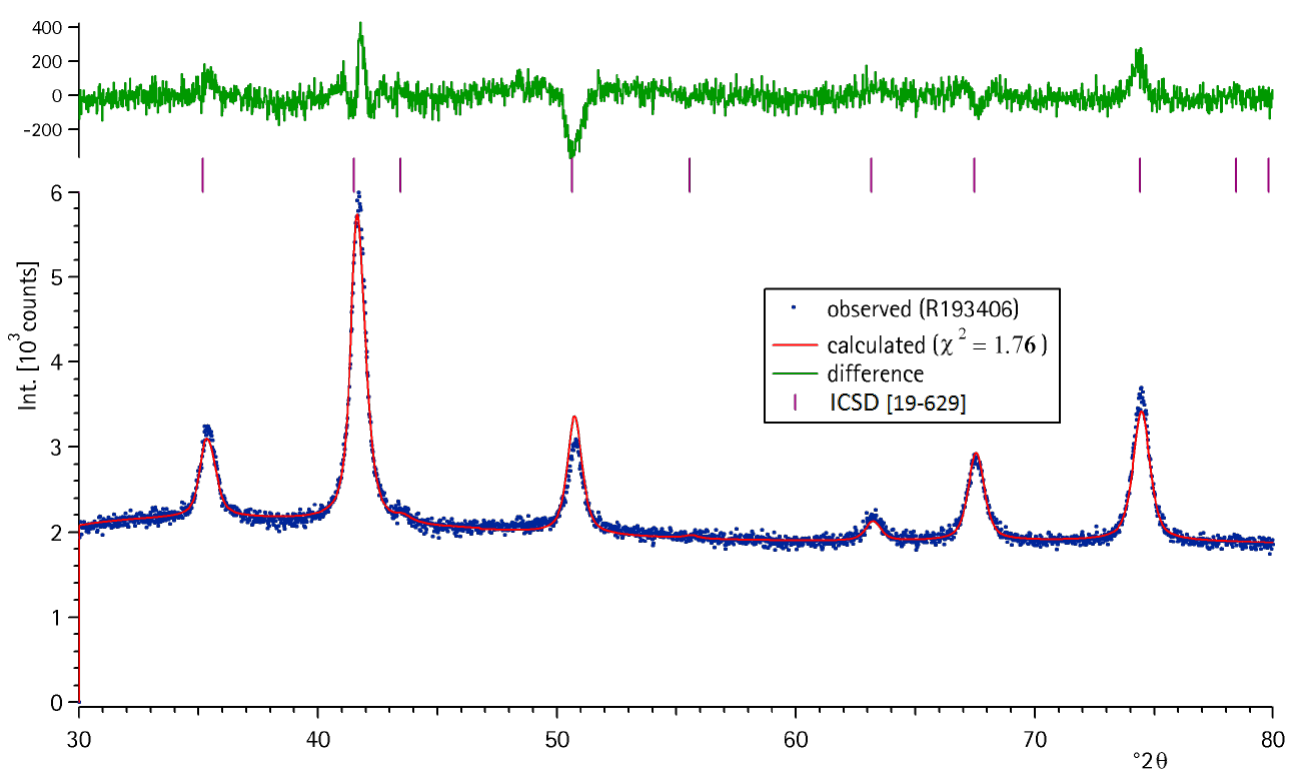
PXRD pattern of magnetite fitted with a reflection set of magnetite to determine crystallite size.

The magnetite structure was confirmed by the recorded powder X-ray diffraction (PXRD) patterns (Figure 1) exactly matching with the standard (ICDD card no. 19-629).

**Figure 2:**
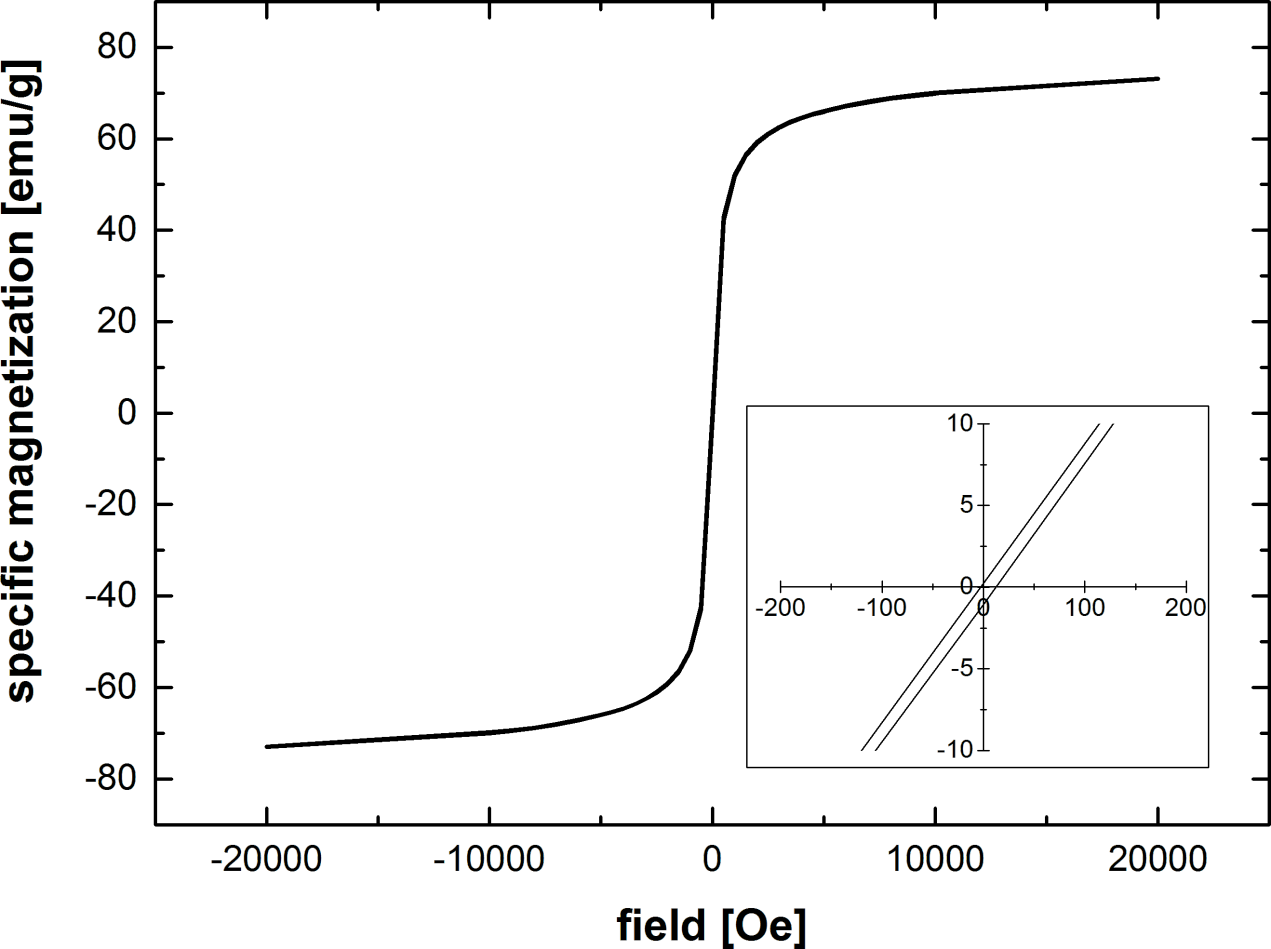
Magnetization curve of 25 nm magnetite particles at 300 K.

The calculated crystal size of 14 nm was smaller than the particle size that could be determined by SEM or BET measurements (data now shown). From nitrogen adsorption a particle size of 24.5 nm was calculated. Observations by scanning electron microscopy (SEM) revealed a particle size of 24 nm (d50; σ = 6 nm). Additionally, the specific (mass dependant) magnetization of the particles was determined at a temperature of 300 K (Figure 2).

### Surface modification of magnetite nanoparticles

Iron oxide nanoparticles were chemically modified by using a combination of citrate and tetramethylammonium hydroxide (TMAH). The positive surface charge of the particles enabled a strong interaction with the negatively charged protein matrix [13] provided by the HSA molecules.

#### Formulation design and characterization of USPIO HSA hybrid particles by dynamic light scattering

Nanoparticles were prepared by ethanolic desolvation in absence and in presence of USPIO. The particle size and zeta potential observed by dynamic light scattering (DLS) measurements significantly increased with the amount of iron oxide particles present during the desolvation process (ANOVA). Particles with the highest content of iron oxide were crosslinked with increasing amounts of glutaraldehyde and a dense particle structure was formed to incorporate the contrast agent.

**Figure 3:**
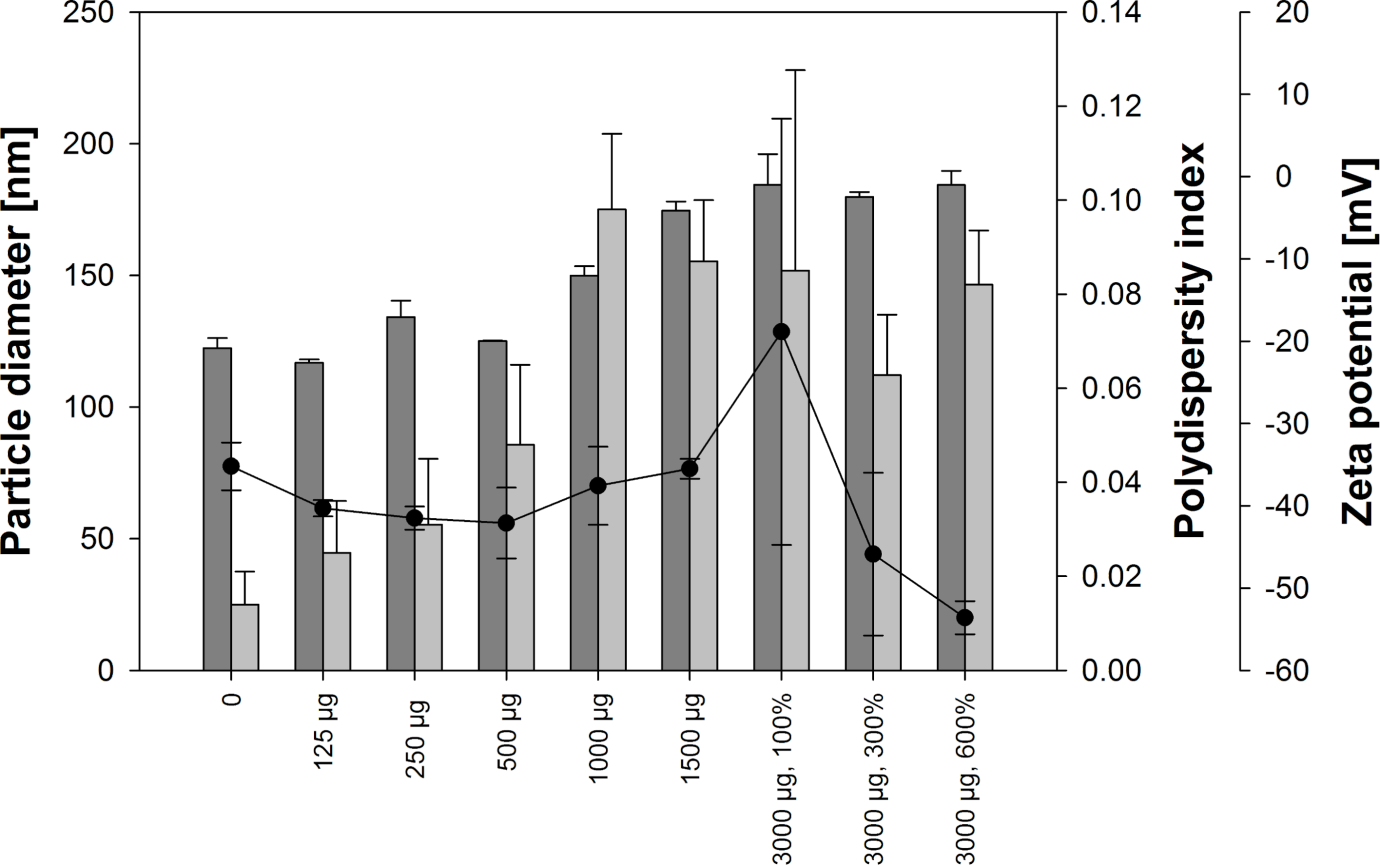
Particle size (dark grey bars), polydispersity (black dots), and zeta potential (light grey bars) of USPIO HSA hybrid particles generated at pH 8.5 at different USPIO concentrations (125–3000 µg per 100 mg) and crosslinker amounts (100–600%), average ± SD, *n* = 3.

A decreasing standard deviation of the polydispersity indicated a narrow size distribution for particles that were crosslinked with higher amounts of glutaraldehyde (Figure 3), even when there were high concentrations of the contrast agent present during desolvation procedure.

Additionally, the influence of the adjusted pH value on particle properties during desolvation was observed. At a pH of 7.5 and 9.5 microparticles with a broad size distribution were generated (data not shown). A pH of 8.5 was optimal for all tested USPIO concentrations.

Interestingly, zeta potential increased with rising amounts of USPIO present during the preparation process. A total value of −20 mV was not exceeded. Since similar values have been reported for stable unmodified HSA particles [15] and drug-loaded nanoparticles earlier [14], these results suggest that the hydrophilic matrix still provides properties identical to those that have been used for drug targeting applications.

#### USPIO load of USPIO HSA hybrid particles

After optimization of formulation design, the drug load of USPIO HSA hybrid particles was determined for particle systems prepared in presence of 10, 20, and 30 µg/mg of HSA. Particle concentration as a mixture of HSA and USPIO encapsulated into the particle matrix was quantified by microgravimetry. Afterwards, the Fe(III) content in the particles was analysed by ion chromatography. USPIO load was calculated as described below (Equation 1).

[1]
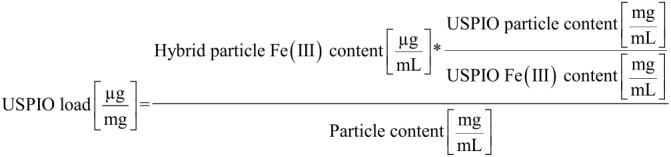


The USPIO load was ranging from 47.39 ± 8.54 µg/mg to 127.14 ± 8.54 µg/mg indicating a washout of HSA during purification procedure. The highest drug load was achieved at USPIO to HSA ratio of 30 µg/mg.

#### Observation of particle shape by transmission electron microscopy

**Figure 4:**
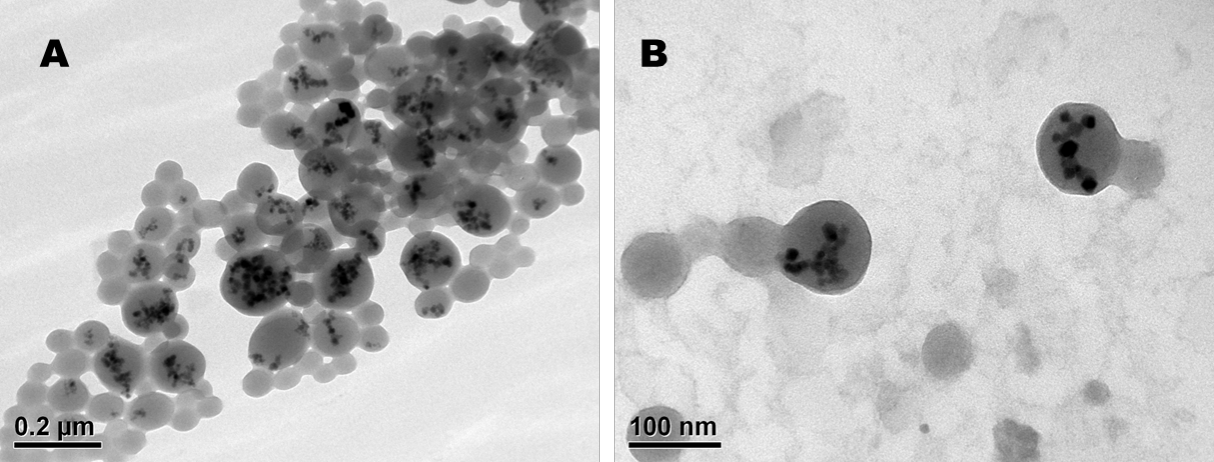
USPIO HSA hybrid particles generated at a USPIO to HSA ratio of 30 µg/mg and crosslinked with glutaraldehyde (100%) in TEM with overview (A) and with perspective on two particles (B).

Electron microscopy of USPIO HSA hybrid particles revealed encapsulation of the modified USPIO into the protein shell (Figure 4).

HSA provides a matrix structure of increasing electron density with increasing amount of crosslinker present during the preparation process. For observations in TEM HSA nanoparticles were chemically stabilized by using the theoretical amount of glutaraldehyde corresponding to a crosslinking of 100% of the 60 amino groups in the HSA molecule.

By this, HSA nanoparticles with transparent appearance (grey) in the TEM were generated. Iron core particles of high electron density appeared in black. Since the agglomeration of HSA molecules during desolvation process is depending on the charge of the molecules, particles increase in diameter with increasing amount of incorporated iron (Figure 4A and B).

#### Determination of particle size distribution by nanoparticle tracking analysis

**Figure 5:**
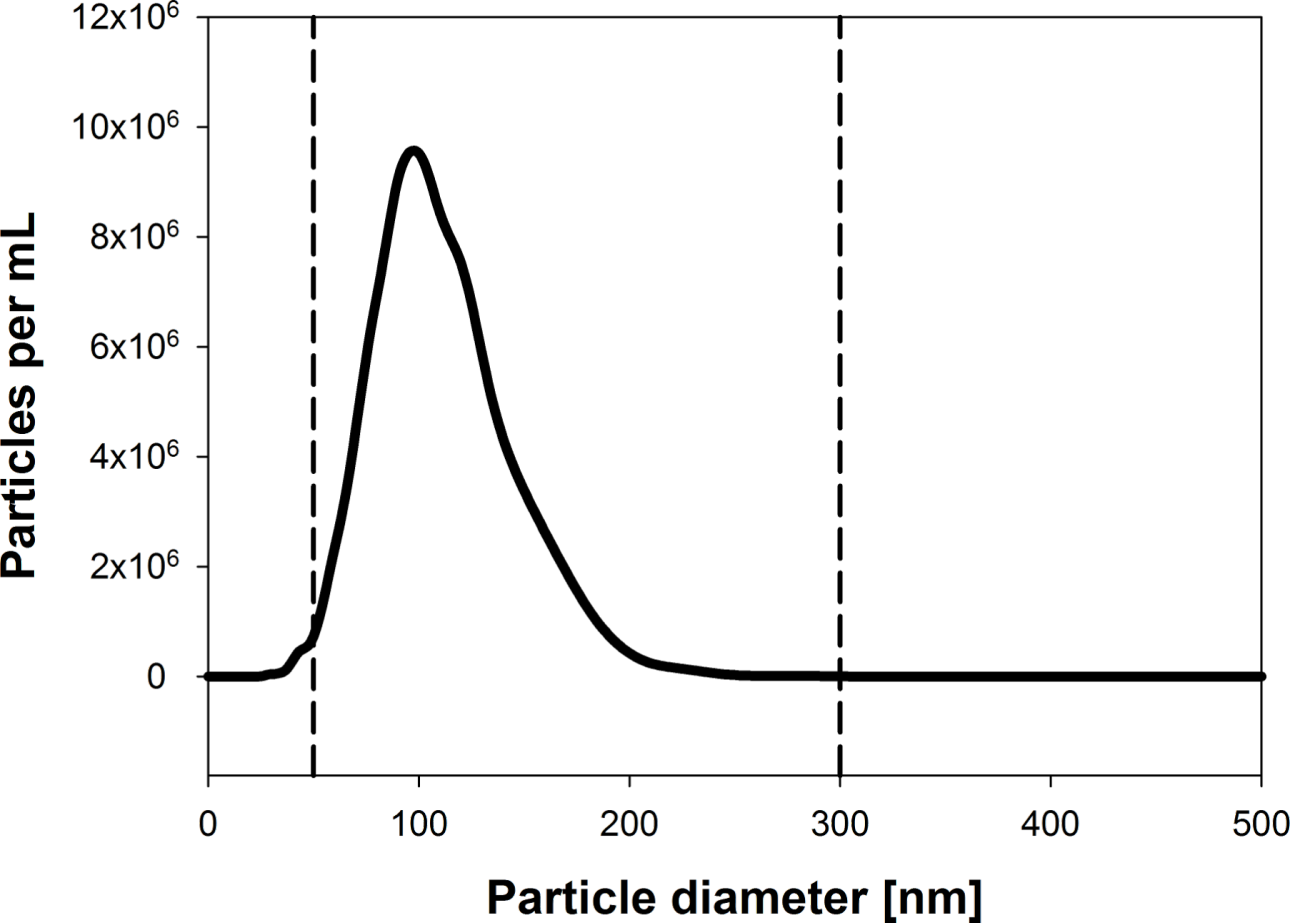
Size distribution of USPIO HSA hybrid particles determined by using NTA with specification range for parenteral application (dotted line, 50–300 nm).

Nanoparticle tracking analysis (NTA) of USPIO HSA hybrid particles generated at a USPIO to HSA ratio of 30 µg/mg revealed a narrow size distribution of nanoparticle suspension with regards to the identified therapeutic application (Figure 5). More than 95% of the particles were in a size range between 50 and 300 nm, that is needed for an optimized accumulation in solid tumors [6]. Therewith, investigations by NTA confirmed earlier observations made by TEM and DLS.

#### Storage stability of USPIO hybrid particles

**Figure 6:**
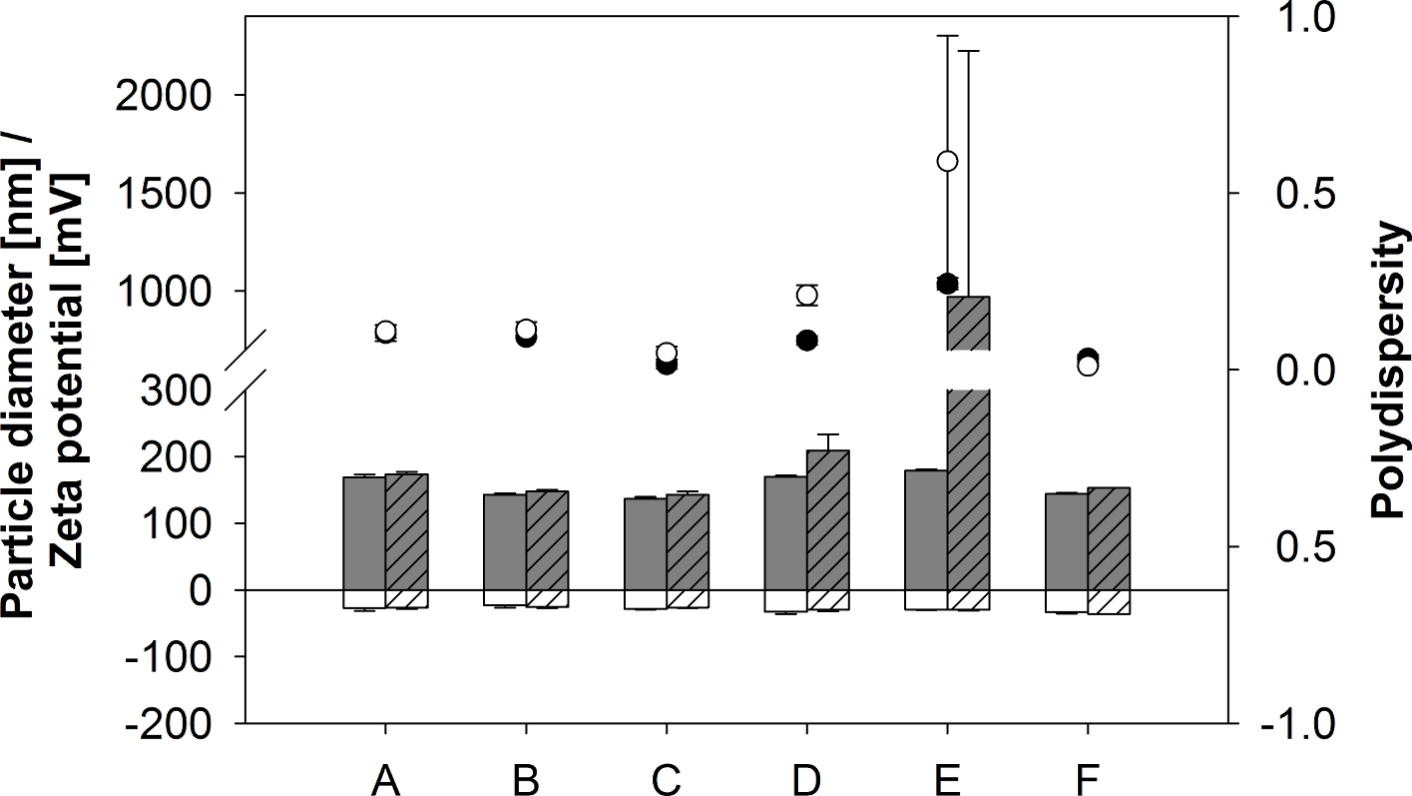
Particle size (grey bars), polydispersity (black dots), and zeta potential (white bars) of USPIO HSA hybrid particles with an USPIO load of 10, 15 and 30 µg/mg in the fridge (A–C) or freeze dried stored at 20 °C (D–F) after 1 day (bars without pattern / black dots), and 2 weeks (bars with pattern / white dots), Average ± SD, *n* = 3.

USPIO HSA hybrid particles with a theoretical drug load of 10, 15, and 30 µg/mg were freeze dried in presence of mannitol at a concentration of 3% (w/v) and tested after 1 day and 2 weeks with regards to their physicochemical properties after resuspension in water (Figure 6). All particle preparations stored under cool conditions remained stable over the evaluated time range. After freeze drying, only USPIO HSA hybrid particles prepared at an iron concentration of 15 µg/mg demonstrated significant agglomeration.

## Experimental

### Reagents and chemicals

Human serum albumin (Fraction V) and glutaraldehyde 8% aqueous solution were purchased from Sigma (Steinheim, Germany). Magnetite particles were friendly provided by Merck KGaA (Darmstadt, Germany). All reagents were of analytical grade and used as received.

### Characterization of magnetite nanoparticles by powder X-ray diffraction

The PXRD pattern of the dried magnetite nanoparticles was measured by using a Stoe StadiP θ–θ-diffractometer (Stoe GmbH, Darmstadt, Germany). Co Kα irradiation was applied. These reflections were used to fit the pattern following observations by Thompson, Cox & Hastings [16–17]. Crystallite size was calculated by using the Scherrer equation [18].

### Magnetization of magnetite nanoparticles

The specific (mass depended) magnetization of the dried magnetite nanoparticles was determined by using a Quantum Design SQUID magnetometer (Quantum Design Inc., San Diego, USA). The sample was fixed in a gelatine capsule and moved through the induction coils (vibrating sample mode) of the magnetometer at constant temperature (300 K) and a series of different constant external fields.

### Surface modification of magnetite 25 USPIO

An amount of 3 g of magnetite 25 particles in isopropanol 20% [v/v] were sedimented magnetically. To the supernatant 26 mL of purified water and 14 mL of salpetric acid 65% [v/v] were added, resulting in a 2 M solution of HNO_3_. This procedure was followed by 3 h of mixing in a roll mixer. The suspension was purified by sedimentation and redispersion in purified water until a neutral pH value was achieved. A volume of 100 mL of an ammonium citrate solution 1% [w/v] was added and the suspension was finally filled up to 500 mL. The suspension was incubated at 70 °C in an ultrasonic bath (Bandelin electronic, Berlin, Germany) and the pH was determined again. The USPIO particle content was determined by microgravimetry. Additionally, the content of Fe(III) was quantified for calculation of the USPIO load.

### Nanoencapsulation of USPIO into human serum albumin

USPIO were incorporated into HSA nanoparticles by the well-known desolvation method [15,19]. In principle, an amount of 50 mg of HSA was dissolved in 10 mM sodium chloride solution. The pH was adjusted to between 7.5 and 9.5. Afterwards, the solution was filtered through a 0.22 μm filtration unit (Schleicher und Schüll, Dassel, Germany). Between 62.5 and 1500 µg of USPIO in aqueous suspension were added and filled up to a volume of 1.0 mL. Ethanolic desolvation was initiated by continuous addition of 4.0 mL of ethanol at a rate of 1.5 mL/min under permanent stirring (550 rpm). Afterwards, a volume of aqueous glutaraldehyde solution 8% [v/v] corresponding to between 100% and 600% of stoichiometric crosslinking of the amino groups in 50 mg HSA was added to stabilize the resulting protein nanoparticles. Particles were stirred for a minimum of 3 h and purified by 3 cycles of centrifugation (16100*g*, 8 min) and redispersion in 1.0 mL water over 5 min in an ultrasonic bath (Bandelin electronic, Berlin, Germany).

### Determination of particle size, size distribution, and surface characteristics

The average particle size, polydispersity, and zeta potential were determined by dynamic light scattering using a Malvern Zetasizer Nano (Malvern Instruments, Malvern, UK). For determination of the zeta potential a Malvern Dip Cell was used. All samples were diluted 1:50 with purified water before the measurement. The nanoparticle content was quantified by microgravimetry.

### Quantification of iron content by ion chromatography

The iron content of USPIO HSA hybrid particles was quantified by using ion chromatography. An amount of 100 mg of particle mass was transferred to a 1.5 mL glass vial. The matrix structure was degraded by addition of 200 µL of hydrochloric acid 37% [w/w] and 50 µL of salpetric acid 65% [w/w]. All samples were incubated over 16 h at room temperature. Afterwards, temperature was increased to 70 °C at a rate of 10 °C/h in a Thermomixer (Eppendorf, Hamburg, Germany) reducing the sample volume to approximately 100 µL. The sample was diluted by a factor of 10 to 30 and analyzed in a Merck–Hitachi Lichrograph system (Merck–Hitachi, Darmstadt, Germany). An IONPAC CS5A (Thermoscientific, Sunnyvale, USA) column was used. The eluent was composed of an aqueous solution of 7 mM pyridine-2,6-dicarboxylic acid, 178 mM potassium hydroxide, 56 mM sulfuric acid, and 74 mM formic acid and pumped at an constant flow rate of 1 mL/min. The PAR reagent contained 0.55 mM 4*-*(2-pyridylazo)resorcinol monosodium salt monohydrate, 1 M 2-(dimethylamino)ethanol, 0.5 M aqueous ammonia solution, and 0.3 M sodium hydrogen carbonate. The flow rate was adjusted to 0.15 mL/min. The UV–vis detector was adjusted to a detection wavelength of 520 nm. From the determined content of Fe(III) the amount of magnetite in the sample was calculated.

### Transmission electron microscopy of USPIO HSA hybrid particles

For observation of particle shape USPIO HSA hybrid particles with an USPIO load of 30 µg/mg and crosslinked with a volume of glutaraldehyde corresponding to a 100% of the amino groups of the HSA molecule were used. All samples were applied to copper grids with a carbon-coated Pioloform film and dried overnight at room temperature. The morphology of the particles was investigated with a Philips EM 208S electron microscope, at a nominal magnification of 16,000–21,000 and analyzed by using the GATAN software package (Gatan Inc., Pleasanton, USA).

### Particle size distribution by nanoparticle tracking analysis

NTA was conducted by using a NanoSight LM20 (NanoSight, Amesbury, United Kingdom), equipped with a sample chamber with a 640 nm laser and a Viton fluoroelastomer O-ring. USPIO-HSA hybrid particles with an USPIO load of 30 µg/mg were injected in the sample chamber with sterile syringes. All measurements were performed at room temperature. The software used for capturing and analyzing the data was the NTA 2.3 (Build 2.3.5.0033).

### Freeze drying of USPIO HSA hybrid particles

For the drying process, 3% (w/v) of mannitol were added to a particle suspension containing 5 mg of nanoparticles (theoretical drug load of 10, 15, 30 µg/mg) and a final volume of 3 mL was adjusted. The formulations were put into a Christ Epsilon 2–7 freeze dryer (Christ, Osterrode, Germany). A slightly modified version of the freeze drying protocol described by Wacker et al. [15] has been used. Initially, product temperature was reduced from +20 to −60 °C and samples were kept under these conditions for 1 h. Primary drying was initiated by evacuating the chamber to a pressure of 0.940 mbar and increasing the temperature to −30 °C over 150 min. Further reduction of pressure (0.006 mbar) was undertaken in conjunction with an increase in temperature to −10 °C over 60 min and conditions were maintained over a time frame of 35 h. For secondary drying the temperature was finally increased to 10 °C over 60 min and to 20 °C over 600 min.

### Storage stability of USPIO HSA hybrid particles

For examination of storage stability the freeze dried samples were stored at 20 °C and resuspended in 3.0 mL of purified water after 1 day and 2 weeks. Particle size, size distribution and zeta potential were determined as described previously. Liquid particle preparations of the same composition were stored in the fridge over the same time period and tested for particle integrity at the same time points.

### Statistical analyses

Particle size, polydispersity indices, and zeta potential data was statistically analyzed with Sigmaplot 11 (Sysstat Software, San José, USA). ANOVA at a significance level of α = 0.05 was applied. All other calculations were carried out by using Excel^©^ 2003 (Microsoft, Redmond, WA, USA).
